# Access and use of WHO essential medicines in Italy

**DOI:** 10.3389/fpubh.2023.1211208

**Published:** 2023-10-10

**Authors:** Alessandro Petrella, Filomena Fortinguerra, Agnese Cangini, Andrea Pierantozzi, Francesco Trotta

**Affiliations:** Italian Medicines Agency, Rome, Italy

**Keywords:** essential medicines, national formulary, health policy, drug consumption, expenditure

## Abstract

**Background:**

Many countries use the WHO Essential Medicines List (EML) as a guide for health policy choices to promote the efficient use of healthcare resources or adopt the concept of essential medicines (EMs) to develop their own national list of essential medicines. The aim of this study is to analyse the availability and use of medicines included in the 22nd WHO EML in Italy.

**Methods:**

Using the ATC code (5th level), a comparison was made between the medicines included in the WHO EML and those retrieved from the Italian Medicines Agency (AIFA) database. The availability (regulatory and reimbursement status) of EMs, as well as the market share in expenditure (million euros) and consumption [measured in WHO-defined daily doses (DDDs)], compared to all reimbursed medicines in 2021, were analysed.

**Results:**

In 2021, approximately 85.2% (*n* = 414) of medicines included in the WHO EML were commonly marketed in Italy. Of these, 396 EMs were fully reimbursed by the Italian National Healthcare Service (INHS), corresponding to 81.5% (396/486) of the WHO EML, while the remaining 18.5% (90/486) were neither authorised (*n* = 72) nor reimbursed (*n* = 18). The study found a low coverage for anti-parasitic, insecticides, and repellent products (ATC P) in addition to medicines for the genitourinary system and sex hormones (ATC G). Even though medicines on the WHO EML, including therapeutic alternatives, accounted for ~48.5% of the expenditure for medicines reimbursed by INHS, the list covered 74% of all national drug consumed. Novel high-cost therapies indicated in high-prevalence diseases and rare conditions, mostly antineoplastic and immune-modulating agents (ATC L) not included in the WHO EML, were also guaranteed.

**Conclusions:**

In Italy, high coverage of EMs was found. It was largely reimbursed by the INHS, even when compared to other European countries. Essential medicines represented a high percentage of the overall expenditure and consumption in Italy. The WHO EML could be an important tool to guide the health policy choices of high-income countries, although a more frequent update and easier access to information on rejected medicines are needed.

## Background

In 1977 the World Health Organisation (WHO) created the first list of essential medicines (EML). The list defines medicines satisfying the priority healthcare needs of a country's population, which are selected on the basis of the high prevalence of a disease and its public health relevance. It includes appropriate dosage forms, robust evidence regarding clinical efficacy and safety, comparative costs (which will vary locally due to the different pricing policies), and cost-effectiveness (1). Essential medicines are identified to extend the primary healthcare coverage of a population. It takes into consideration the health situation of developing countries with the aim of making therapies widely available and accessible in their national health systems (2). Since then, the list has been used as a good starting point to develop a voluntary national formulary or national essential medicines list (NEML) in many low- and middle-income countries (LMICs) to guide the health policy choices of governments and relevant institutions to meet the primary needs of their healthcare systems (3). The list may also be considered a tool for developed countries to support national medicine policies while promoting the efficient use of healthcare resources by selecting medicines having an added therapeutic value over the multitude of commercially available alternatives and thus reputed to be essential for human health (4–8). Finally, the list could also be used as a support for clinicians and physicians in the process of deciding medicines for prescription, thereby promoting their appropriate use (9).

The WHO EML is updated and published every 2 years by the WHO Expert Committee on Selection and Use of Essential Medicines. The 22nd edition of the WHO EML (10), updated on September 2021, contains over 500 medicines selected as a result of evidence-based criteria as the most effective and safe and that should be widely available to patients at affordable prices. They are identified by active ingredient and listed together with their intended use, strength, and formulation, organised in chapters dealing with specific pharmacological classes or diseases (10).

Since a NEML is intended to meet the needs of its population, countries that are geographically close or similar to each other in population size, health status, life expectancy, and healthcare expenditure might be expected to have similar lists (11).

Though several articles are published worldwide on access to medicines, few publications provide information on the availability of essential medicines (EMs) in high-income countries (HICs) (12–14). One study, which systematically compared the NEMLs of 137 countries, including 21 HICs (with the exception of Italy), showed substantial gaps in the selection of medicines at the national level compared with those recommended by the WHO EML (12). In particular, 23 countries (17%), including some European countries (e.g., Sweden, Portugal, Malta, Poland, Czechia, Slovakia, and Slovenia), have 50% lower coverage with 2017 WHO EML, while 27 countries (20%), mostly LMICs, have at least 80% coverage. Countries with lower health expenditure, such as Angola (*n* = 13) and Cambodia (*n* = 9), appear to have omitted from their lists a larger number of medications listed in the WHO EML. However, countries with higher health expenditure appear to have included more medicines in their lists that are not on the WHO EML—leading among them are Slovakia (*n* = 692) and Portugal (*n* = 649), with the exception of Sweden (*n* = 146). The inclusion of additional medicines in HIC national lists beyond those listed in the WHO EML could explain the difference in coverage (expressed as a percentage of EMs overall included) compared to LMIC national lists, where the total number of medicines included is very low.

We are not aware of any studies published on this topic referring to the Italian context. Italy has a universal health service, and decisions relating to coverage and pricing of medicines reimbursed by the Italian National Health Service (INHS) are determined at the national level by the Italian Medicines Agency (AIFA). The aim of this study is to assess the availability of EMs in Italy and to analyse the market share of EMs in terms of consumption and expenditure in 2021, in addition to highlighting other medicines not included in the WHO list but fully reimbursed in Italy.

## Methods

The 22nd edition of the EML (10) was used to extrapolate the following information on medicines: Anatomical Therapeutic Chemical (ATC) 5th level, active ingredients, dosage forms (strength and numbers of units), routes of administration, therapeutic indications, and alternatives (where reported). These records were linked by ATC code (5th level) to the AIFA database, which included the following variables: (i) WHO-defined daily doses (DDD) included in the package; (ii) regulatory status in Italy (authorised or withdrawn); (iii) availability in the Italian market; and (iv) coverage status (reimbursed or not reimbursed). This database was then linked to Italian pharmaceutical administrative databases, which provided information at the package level on consumption and expenditure for all drugs marketed in Italy in 2021.

When the ATC (5th level) was not present in the AIFA database, we searched for the active ingredient in order to include in the analysis those medicines available in Italy with a different ATC 5th level for the same indication, as in the case of some monovalent vaccines (e.g., measles vaccine, pertussis vaccine) that are registered in Italy as polyvalent vaccines.

For the second objective of the study, we analysed the market share of EMs in consumption and expenditure in 2021. To do this, we aggregated all expenditure and consumption values of ATCs (5th level) included in the AIFA database into the following four groups:

- Group 1: EMs, consisting of all medicines included in the WHO EML and available in Italy;
- Group 2: WHO alternatives, including all medicines (ATCs 5th level), reported as therapeutic alternatives within the WHO EML list and available in Italy;
- Group 3: Analogues, comprising all medicines with the same ATCs (4th level) as those in the WHO EML (including WHO alternatives) available in Italy (net of the WHO EML and their reported alternatives). Antineoplastic and immune-modulating agents (ATC L) were excluded from this analysis, as analogues in this category cannot be identified by applying directly the ATC 4th level;
- Group 4: Others, including the remaining medicines (ATC 5th level) retrieved from the AIFA database and marketed in Italy and not included in the previous three groups.

For each group, we estimated the consumption (DDD), expenditure, and average cost per DDD in 2021. In addition, we estimated the proportion of expenditure and consumption for each group in relation to total expenditure and consumption to determine the coverage of WHO EMs or related analogue EMs (ATC 4th level). All medicines included in the analyses were identified by active ingredient (medicine) and not by medicinal product.

## Results

### Coverage of WHO EML in Italy

**Figure 1:**
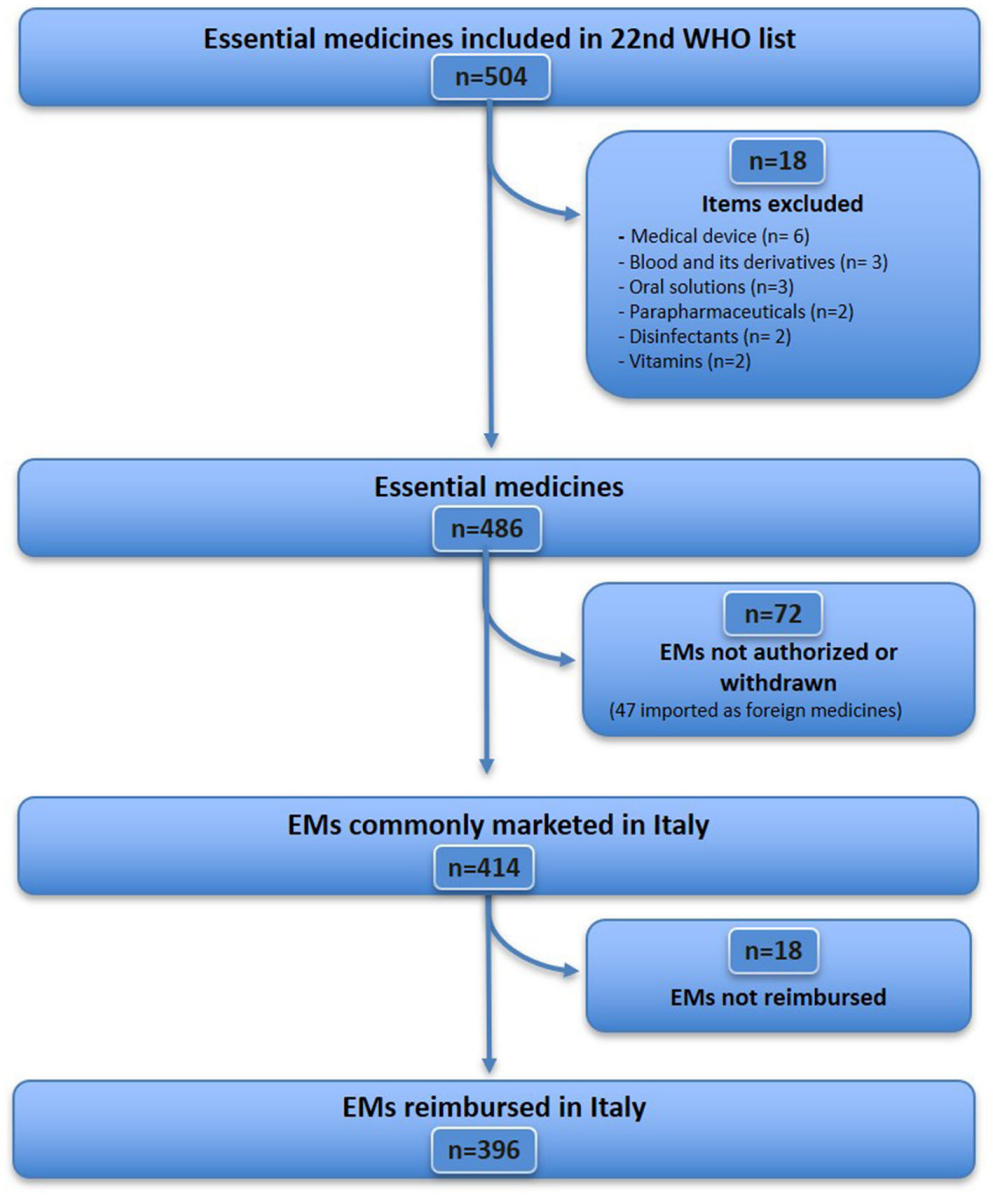
Availability of WHO essential medicines in Italy.

**Figure 2:**
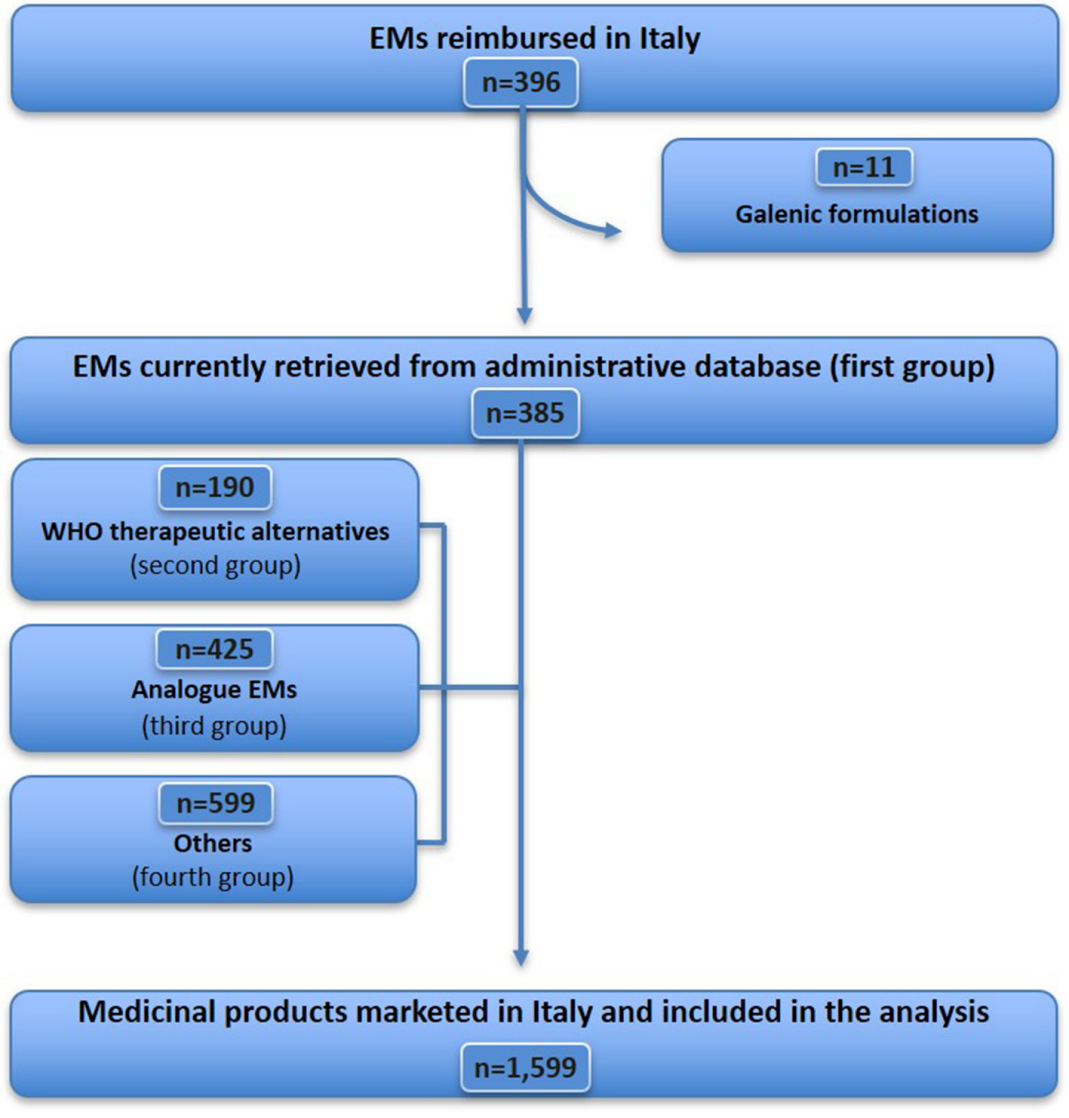
Medicines included in the expenditure and consumption analysis in Italy in 2021.

The WHO EML (22nd edition) includes 504 items (Figure 1), of which 18 were excluded because they did not fit the definition of medicine (see Supplementary Table S1 for details), resulting in a sample of 486 essential medicines considered for analysis. Further, 72 EMs (from 486) were excluded because they were unavailable [although some of them were imported as foreign medicines (*n* = 47), Supplementary Table S2], unauthorised, or withdrawn in Italy (*n* = 25) (Supplementary Table S3). Therefore, 414 EMs that were commonly marketed in Italy were identified, accounting for 85.2% (414/486) of the WHO EML list. Of these, 18 medicines were not reimbursed in Italy (Supplementary Table S4). A total of 396 EMs were fully reimbursed in Italy, corresponding to 81.5% (396/486) of the WHO EMs list. For the analysis of expenditure and consumption (Figure 2), 11 out of 396 medicines were excluded, as they are available only as galenic formulations and, therefore, could not be found in the Italian administrative databases (Supplementary Table S5), resulting in a total of 385 WHO EMs, which represented the first group considered in the analysis of expenditure and consumption.

For each EM retrieved from the Italian administrative databases, we identified the therapeutic alternatives indicated by the WHO, totalling 190 medicines (ATC 5th level) available in the Italian market in 2021, representing the second group. Additionally, based on the medicines belonging to the first and second groups, a total of 425 analogues, i.e., medicines having the same ATC (4th level) as EMs, were identified, representing the third group. Finally, the remaining medicines marketed in Italy but not included in the previous groups (599 medicines for ATC 5th level) were categorised under the fourth group (Supplementary Table S6). For expenditure and consumption analysis, we considered these four groups comprising a total of 1,599 medicines (by ATC 5th level) marketed in Italy in 2021.

**Figure 3:**
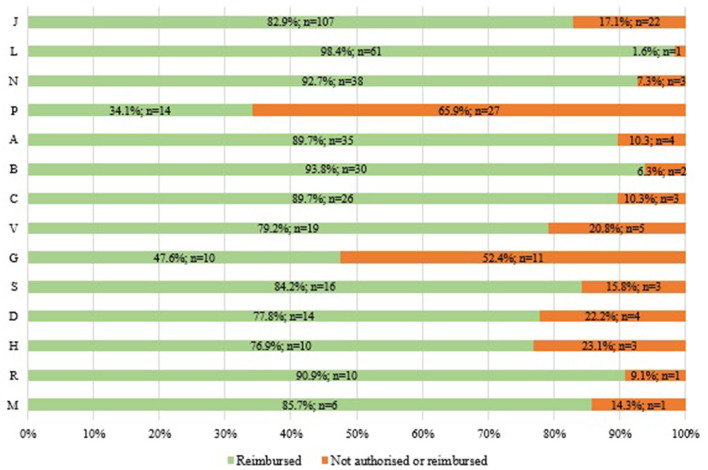
Reimbursement status of WHO EMs by ATC (1^st^ level) in Italy in 2021 (*N* = 486: 396 reimbursed and 90 not authorised or reimbursed).

According to our analysis of the reimbursement status of WHO EMs in Italy by ATC (1st level) 2021 (*n* = 486) (Figure 3), we found that there is low coverage for anti-parasitic, insecticides, and repellent products (ATC P: 34%) as well as medicines for the genitourinary system and sex hormones (ATC G: 48%). On the other hand, we found a high coverage for antineoplastic and immuno-modulating agents (ATC L: 98%). We also found coverage of <80% for systemic hormone preparations, excluding sex hormones and insulins (ATC H: 77%), dermatological products (ATC D: 78%), and miscellaneous (ATC V: 79%).

**Figure 4:**
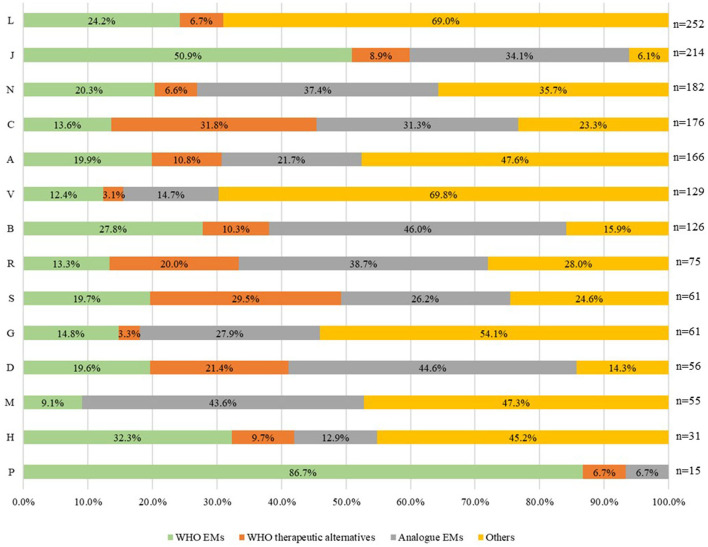
Distribution of the four groups of medicines reimbursed in Italy in 2021 by ATC (1st level) (*N* = 1,599).

When analysing the distribution of the four selected groups of reimbursed medicines (*n* = 1,599) by ATC (1st level) for 2021 in Italy (Figure 4), we found that anti-parasitic, insecticides, and repellent products (ATC P) had the highest relative frequency of WHO EMs accounting for 87% EMs and 7% WHO therapeutic alternatives. Anti-infectives for systemic use (ATC J) accounted for 51% of EMs and 9% of WHO therapeutic alternatives), while medicines for sensory organs accounted for 19.7% of EMs and 29.5% of WHO therapeutic alternatives. Medicines for the cardiovascular system (ATC C) accounted for 13.6% of EMs and 31.8% of WHO therapeutic alternatives. The first two categories (ATC P and ATC J) also had the lowest percentage of “others” reimbursed, 6.7% and 6.1% respectively. On the other hand, antineoplastic and immune-modulating agents (ATC L) and miscellaneous (ATC V) were the ATC groups with the highest percentage of “others” reimbursed (69%). Medicines for blood and blood-forming organs (ATC B) had the highest percentage of analogues reimbursed (46%), including, for example, recombinant blood coagulation factors or antiplatelet agents, such as ticagrelor. ATC L was excluded from the third group represented by analogues (ATC 4th level).

### Expenditure and consumption analysis

In 2021, the public expenditure for the four medicine groups considered in the analysis was €23,465 million, corresponding to a total of 27,734 million DDD consumed (Table 1); 28.4% of the expenditure (€6,668 million) was related to WHO EMs commercially available in Italy and accounted for 40.4% (11,198 million DDD) of the total national consumption of medicines reimbursed by INHS. When therapeutic alternatives indicated by WHO were added to the EMs, the expenditure and consumption figures increased, reaching 48.5% and 73.8% of the total amounts, respectively and represented the coverage of WHO EML expenditure and consumption in Italy in 2021.

**Table 1:** Group-wise expenditure and consumption of reimbursed medicines in Italy in 2021.

**Group**		**Medicines**	**Expenditure (million euros)**			**DDD (million)**			**Average cost per DDD (euros)**
		**N**.	**N**.	**%**	**% cum**	**N**.	**%**	**% cum**	
1	WHO EMs	385	6,668	28.4	28.4	11,198	40.4	40.4	0.59
2	WHO therapeutic alternatives	190	4,724	20.1	48.5	9,284	33.5	73.8	0.51
3	Analogue EMs (ATC 4^th^ level)^*^	425	3,709	15.8	64.0	4,169	15.0	88.9	0.87
4	Others	599	8,362	35.6	100	3,081	11.1	100	2.72
Overall		1,599	23,465	100	-	27,734	100	-	0.85

^*^Antineoplastic and Immunomodulating Agents (ATC L) were excluded.

If the analysis extended to medicines with the same ATC (4th level) as those included in the WHO EML (named analogue EMs), excluding antineoplastics and immunomodulators, then the total expenditure and consumption reached 64% and 88.9%, respectively. The expenditure for the medicines categorised under the “others” group was €8,362 million (35.6% of total expenditure), while consumption was approximately 3,100 million DDD, covering only 11.1% of total national drug consumption.

The average cost per DDD for the WHO EMs was the lowest, ranging from €0.59 for EMs to €0.51 for the WHO therapeutic alternatives, and accounted for 73.9% of the total consumption and only 48.4% of the expenditure. The average cost for analogues (ATC 4th level) of EMs (including alternatives) was €0.87, 70% higher than that of WHO EMs, while the cost of “others” was the highest at €2.72, which was five times higher than the cost of WHO EMs. The last two groups (analogues and “others”) accounted for 26.1% of total consumption and 51.4% of expenditure.

**Figure 5:**
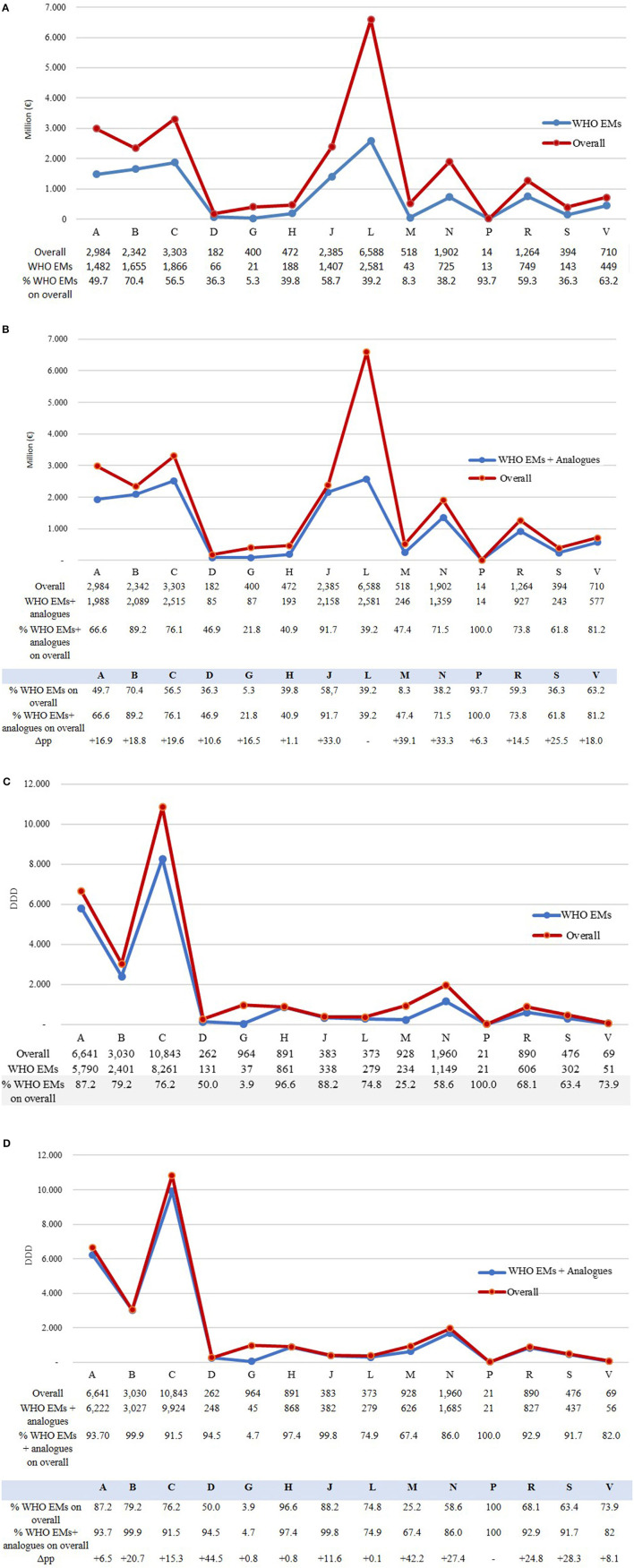
**(A–D)** Expenditure (million euros) and consumption (measured in DDD) of WHO EMs (including alternatives) and analogue ATCs (5th level) by ATC (1st level) compared to the overall of medicines reimbursed in Italy in 2021.

Figure 5 compares the expenditure and consumption of the overall medicines reimbursed (by ATC 1^st^ level) in Italy in 2021 by categorising them into two groups. One group included only the WHO EMs and alternatives (Figures 5A, C), and the other group included the WHO EMs and alternatives along with analogue ATCs (5^th^ level) (Figures 5B, D). An analysis of the proportion of expenditure (Figures 5A, B) by drug category (ATC 1st level) of medicines included in the WHO EML, including alternatives against the total expenditure for reimbursed medicines in Italy, indicates that the lowest amount was spent on medicines for the genitourinary system and sex hormones (ATC G: 5.3%) and medicines for the musculoskeletal system (ATC M: 8.3%) (Figure 5A). This suggests low coverage or a higher gap compared to the total expenditure on WHO EMs in both these categories. However, anti-parasitic, insecticides, and repellent products (ATC P: 93.7) and medicines for blood and blood-forming organs (ATC B: 70.4%) had the highest spending and coverage. By adding analogues to ATC 4th level (Figure 5B), the coverage increased for all drug categories (ATC 1st level), thus reducing the gap between WHO EMs and total reimbursed medicines in Italy. Overall, with the addition of analogues, ATC P (100%), ATC J (91.7%), ATC B (89.2%), and ATC C (76.1%) had the highest coverage in expenditure compared to the total. Three categories registered a significant increase mostly because they had many analogues—medicines for the musculoskeletal system (ATC M) increased from 8.3% to 47.4% (+39.1 percentage points), medicines for the central nervous system (ATC N) from 38.2% to 71.5% (+33.3 percentage points), and anti-infectives for systemic use (ATC J) from 58.7% to 91.7% (+33.0 percentage points). However, systemic hormonal preparations, excluding sex hormones and insulins (ATC H: +1.1 percentage points) and anti-parasitic products, insecticides, and repellent products (ATC P: 6.3 percentage points), showed the lowest variations.

Analysing drug consumption (Figures 5C, D) by ATC (1st level), the share of WHO EML (including alternatives) in total reimbursed medicines was high for all categories, suggesting a high coverage of WHO EMs in drug consumption across all categories (Figure 5C), with the exception of dermatological products (ATC D: 50.0%), medicines for the musculoskeletal system (ATC M: 25.2%), and genitourinary system and sex hormones (ATC G: 3.9%).

However, even for consumption, when the ATC 4th level analogues (Figure 5D) were added, coverage was found to be high (>90%) for most categories, and it increased especially for dermatological products (ATC D: +44.5 percentage points) and medicines for the musculoskeletal system (ATC M: +42.2 percentage points) but not for genitourinary system and sex hormones (ATC G) and systemic hormonal preparations. However, within this group, sex hormones and insulins (ATC H) registered a slight increase (+0.8 percentage points) in coverage, thus maintaining a huge gap between the WHO EMs and the total consumption in Italy. With regards to ATC G, medicines for benign prostatic hyperplasia (tamsulosin, alfuzosin, finasteride, silodosin, and terazosin) and hormone replacement therapy in postmenopausal women (estriol, estradiol, and tibolone) were included in the AIFA database but not in the EML by WHO.

If we consider the “others” group that included medicines reimbursed by the INHS but not listed WHO's EML and analogues, we found that they were primarily represented by novel high-cost treatments, such as antineoplastic agents, medicines targeting small population subgroups, or medicines for rare diseases (Supplementary Table S6). Antineoplastic and immuno-modulating agents (ATC L) comprised 25% of drugs belonging to the “others” group (Figure 5D) and accounted for more than 60% (4 million euros) of the total expenditure of this category (Figure 5B), while the EMs covered 75% of the overall consumption of this category (Figure 5D), with an expenditure coverage of 39.2% (2.5 million euros) (Figure 5B).

## Discussion

This study is the first to analyse the status of EMs in Italy. It compares the Italian drug formulary to the WHO EML, taking into consideration the number of medicines reimbursed by the INHS, as well as national medicines expenditure and consumption. The results highlight the favourable outcome of registration and commercial availability of EMs in Italy. For instance, 81.5% (396/486) of the medicines included in the WHO EML were reimbursed by the INHS, while only 18.5% (90/486) were either not authorised (*n* = 72) or not reimbursed (*n* = 18). This is particularly true when comparing Italy with other developed European countries such as Sweden, Portugal or Malta, where the coverage rates are much lower (Sweden: 49%; Malta: 40%; Portugal: 28%) (12).

The study found a low coverage for anti-parasitic, insecticides, and repellent products (ATC P) and medicines for the genitourinary system and sex hormones (ATC G). It should be noted that some of the drugs belonging to ATC P and not available in Italy are indicated for the treatment of neglected diseases, which are highly prevalent in tropical and subtropical regions (i.e., antimalarial medicines or medicines for the treatment of American and African trypanosomiasis); if necessary, they are provided by INHS by importing them from abroad. Within ATC G, oral contraceptives are available in Italy and purchased by citizens; however, their expenses might be reimbursed to specific population subgroups in some Italian regions (15, 16).

Although the WHO's list (including therapeutic alternatives) represented only 25% of the medicines included in the AIFA database [396 ATCs out of 1,599 ATCs (5th level)], it covered nearly 74% of total drug consumption (measured as DDD) in Italy. This proved that EMs are mostly used in Italy despite the wide availability of other active ingredients. These results were consistent with the WHO's definition of “essential medicine” (2) and confirmed the public health importance of using medicines selected after a systematic evidence-based approach and careful evaluation of efficacy, safety, and cost-effectiveness.

However, many “other” medicines, although not included in the WHO EML, have been reimbursed in Italy. In fact, recent anticancer drugs (e.g., ribociclib and palbociclib for the treatment of breast cancer, osimertinib and alectinib for lung cancer, dabrafenib for the treatment of melanoma), onco-haematologic drugs (e.g., daratumumab), biologic disease-modifying anti-rheumatic drugs (e.g., eculizumab, ustekinumab, and secukinumab), and multiple sclerosis drugs (alemtuzumab, natalizumab, daclizumab, ocrelizumab, and teriflunomide) that are reimbursed in Italy are not included in the EML. For instance, the WHO Expert Committee on the Selection and Use of Essential Medicines did not list disease-modifying medicines for multiple sclerosis until 2022. This brings into question the superiority of the evaluated medicines over other therapeutic options in terms of benefits, harms, and affordability, although a subsequent reassessment of a revised application for EML inclusion would be taken into consideration (17). However, it should be noted that the new WHO EML, updated in July 2023 (23rd edition), included a new section for this therapeutic area (18).

A similar situation was observed for medicines for enzymatic replacement therapy (ERT), a high-cost treatment for a small group of patients with rare congenital diseases. Even for therapeutic categories widely covered by the list of essential drugs, we found several recent therapeutic options among “others.” For example, in this group, we found medicines for the treatment of chronic diseases, such as blood glucose-lowering drugs, excluding insulins, such as glucagon-like peptide-1 (GLP-1) analogues (e.g., dulaglutide and semaglutide) and dipeptidyl-peptidase-4 (DPP-4) inhibitors (e.g., sitagliptin and linagliptin). These findings reveal that the INHS ensures universal access to many innovative, high-cost treatments for all patients, including those with rare diseases.

When analysing the group of medicines considered “analogues” to WHO EMs (including alternatives), we found lower consumption and expenditure shares (~15% of the total), even if the cost per DDD was higher than EMs. However, the presence of analogue medicines as an alternative to EMs, albeit at a higher cost, may be considered acceptable, as it could help address the frequent phenomenon of drug shortages.

Further, when analogues were added to WHO EMs during the analysis, the coverage in terms of consumptions was even higher (>90%) for most categories, with the exception of ATC G, which had a higher gap when the WHO EMs and analogues consumption combined were compared to the total consumption for the same category. For example, medicines for benign prostatic hyperplasia (tamsulosin, alfuzosin, finasteride, silodosin, and terazosin) and hormone replacement therapy in postmenopausal women (estriol, estradiol, and tibolone) were included in the AIFA database but not in WHO's EML, suggesting that these medicines may probably not be considered “essential” to the public health.

This study aimed to describe the availability of WHO EMs in a country with universal healthcare and analyse the expenditure and consumption of these drugs compared to other commercially available drugs. Due to the increased demand and availability of high-cost innovative medicines, universal healthcare systems have been facing significant financial challenges impacting their sustainability in recent decades. From this perspective, the WHO EML can be a valuable tool in promoting efficient use of economic resources, thereby curbing the rise in pharmaceutical expenditures (14). However, the situation has become increasingly complicated due to the rising prices of new drugs that are entering the market and subsequently being selected for inclusion in the WHO EML (19). For example, WHO has included some high-cost innovative medicines, such as immunotherapies for cancer and antivirals for hepatitis C, to the list because of their efficacy, but these medicines are generally unaffordable in most areas of the world. The inclusion of expensive but highly effective medicines in the WHO EML has sparked a heated debate even in high-income countries (HICs), reinforcing the argument that high-cost medicines pose a challenge to all healthcare systems, regardless of the country's economic status (20, 21). On the other hand, since expensive patented medicines are not necessarily excluded from the list, some questions have been raised on the true meaning of “essential” for developed national healthcare systems and patients (4, 19). The inclusion of a particular medicine on the list would seem to indicate that it is very “necessary” for patients, and therefore, the goal would be to proactively promote its access and affordability regardless of its cost. Based on this logic, the WHO EML revision process must be significantly improved to facilitate easy access to innovative medicines that demonstrate clear clinical benefits. Further, providing national decision-makers with easier access to the list of rejected or cancelled medicines, along with their respective reasons and supporting evidence, could be helpful.

This study has the important merit of having assessed not only the registration status of EMs in Italy but also their current availability in the Italian market by consulting several administrative databases. Nevertheless, it has some limitations. First, the expenditure and consumption analysis was conducted by comparing the ATCs included in the WHO EML and the AIFA database, without considering the formulation, dosage, and therapeutic indications recommended by the WHO, but by referring to all diseases and conditions that the selected medicinal products were intended to treat, regardless of whether they were recommended by the WHO. Second, the data on medicines marketed in Italy refer to a single year–2021; the basket of drugs compared could be different in another year. Third, because it was not possible to have a list of rejected medicines, we were unable to evaluate the availability of some medicines considered “non-essential” by WHO.

## Conclusions

The issue of medicinal accessibility has been widely debated in Europe. In Italy, almost all medicines on the WHO EML are available and primarily reimbursed by the INHS. In this context, the EML is a useful tool to support and promote the sustainability of healthcare systems, even in countries that offer advanced universal healthcare to their patient population. Many essential medicines are used in clinical practise, which suggests a cost-effective use of the drugs prescribed. Furthermore, despite the high utilisation of drugs included in the list in Italy, a portion of pharmaceutical spending is oriented towards novel high-cost treatments indicated in high-prevalence diseases and rare conditions.

## Data availability statement

The original contributions presented in the study are included in the article/Supplementary material, further inquiries can be directed to the corresponding author.

## Author contributions

FT contributed to the study's conception and design. APi advised on study methods and analyses. APe performed data collection and data analyses. APe, AC, and FF drafted the manuscript. All authors contributed to the interpretation of data, read, commented, revised, and approved the final version of the manuscript for publication.

## References

[1] Kar SS Pradhan HS Mohanta GP. Concept of essential medicines and rational use in public health. Indian J Community Med. (2010) 35:10–3. 10.4103/0970-0218.62546 20606912 PMC2888334

[2] WHO Expert Committee on the Selection of Essential Drugs World Health Organization. (1977). The Selection of Essential Drugs: Report of a WHO Expert Committee [Meeting Held in Geneva from 17 to 21 October 1977]. World Health Organization. Available online at: https://apps.who.int/iris/handle/10665/41272 (accessed September 7, 2023).

[3] World Health Organization. Promoting Rational Use of Medicines: Core Components. (2002). World Health Organization. Available online at: https://apps.who.int/iris/handle/10665/67438 (accessed September 7, 2023).

[4] Duong M Moles RJ Chaar B Chen TF. World hospital pharmacy research consortium (WHoPReC). Essential medicines in a high income country: essential to whom? PLoS ONE. (2015) 10:e0143654. 10.1371/journal.pone.0143654 26650544 PMC4674059

[5] Serafini M Cargnin S Massarotti A Pirali T Genazzani AA. Essential medicinal chemistry of essential medicines. J Med Chem. (2020) 63:10170–87. 10.1021/acs.jmedchem.0c00415 32352778 PMC8007110

[6] Magrini N. XXIV National Seminar: Evaluating the Use and Safety of Medicines: Experiences in Italy. The WHO List of Essential Medicines: What Value for the Rich Countries? Rome: Istituto Superiore di Sanità (ISS) (2015). p. 14–15.

[7] Jeličić Kadić A Žanić M Škaričić N Marušić A. Using the WHO essential medicines list to assess the appropriateness of insurance coverage decisions: a case study of the Croatian national medicine reimbursement list. PLoS ONE. (2014) 9:e111474. 10.1371/journal.pone.0111474 25337860 PMC4206465

[8] Mahmić-Kaknjo M Marušić A. Analysis of evidence supporting the Federation of Bosnia and Herzegovina reimbursement medicines lists: role of the WHO Essential Medicines List, Cochrane systematic reviews and technology assessment reports. Eur J Clin Pharmacol. (2015) 71:825–33. 10.1007/s00228-015-1861-8 25956715

[9] Taglione MS Ahmad H Slater M Aliarzadeh B Glazier RH Laupacis A. Development of a preliminary essential medicines list for Canada. CMAJ Open. (2017) 5:E137–43. 10.9778/cmajo.20160122 28401130 PMC5378503

[10] World Health Organization Model List of Essential Medicines-−22nd List2021. Geneva: World Health Organizations (2021). (WHO/MHP/HPS/EML/2021.02). Licence: CC BY-NC-SA 3.0 IGO. Available online at: https://www.who.int/publications/i/item/WHO-MHP-HPS-EML-2021.02 (accessed April 24, 2023).

[11] Wirtz VJ Hogerzeil HV Gray AL Bigdeli M de Joncheere CP Ewen MA. Essential medicines for universal health coverage. Lancet. (2017) 389:403–76. 10.1016/S0140-6736(16)31599-9 27832874 PMC7159295

[12] Persaud N Jiang M Shaikh R Bali A Oronsaye E Woods H. Comparison of essential medicines lists in 137 countries. Bull World Health Organ. (2019) 97:394–404C. 10.2471/BLT.18.222448 31210677 PMC6560372

[13] Taglione MS Persaud N. Assessing variation among the national essential medicines lists of 21 high-income countries: a cross-sectional study. BMJ Open. (2021) 11:e045262. 10.1136/bmjopen-2020-045262 34380717 PMC8359480

[14] Piggott T Nowak A Brignardello-Petersen R Cooke GS Huttner B Schünemann HJ. Global status of essential medicine selection: a systematic comparison of national essential medicine lists with recommendations by WHO. BMJ Open. (2022) 12:e053349. 10.1136/bmjopen-2021-053349 35144950 PMC8845216

[15] Toscana, Region. Interventi regionali per l'educazione alla salute sessuale e riproduttiva e per l'accesso alla contraccezione gratuita, come modificata dalla DGR n. 394/2019. Deliberation No 1251/2018. Available online at: http://www301.regione.toscana.it/bancadati/atti/Contenuto.xml?id=5357805&nomeFile=Delibera_n.39_del_23-01-2023 (accessed September 7, 2023).

[16] Puglia, Region. Distribuzione gratuita nel consultori familiari, di contraccettivi ormonali inclusi nel Prontuario Terapeutico in fascia “C”. Deliberation No 483/2008. Available online at: https://www.sanita.puglia.it/ricerca_det/-/journal_content/56/25619/educazione-contraccettiva-sessuale (accessed September 7, 2023).

[17] The selection and use of essential medicines: report of the WHO Expert Committee on Selection and Use of Essential Medicines 2019 2019 (including the 21st WHO Model List of Essential Medicines and the 7th WHO Model List of Essential Medicines for Children) (WHO Technical Report Series No. 1021). Geneva: World Health Organization. (2019). Available online at: https://list.essentialmeds.org/files/trs/upzppOSPYB9uERmSypnQpurE1E4KB2E2nUpxTYDh.pdf (accessed September 7, 2023).

[18] The Selection Use of Essential Medicines 2023. Executive Summary of the Report of the 24th WHO Expert Committee on the Selection and Use of Essential Medicines, 24 – 28 April 2023. Geneva: World Health Organization. (2023). Available online at: https://www.who.int/publications/i/item/WHO-MHP-HPS-EML-2023.01 (accessed September 7, 2023).

[19] Hwang TJ Kesselheim AS Vokinger KN. Reforming the world health organization's essential medicines list: essential but unaffordable. JAMA. (2022) 328:1807–8. 10.1001/jama.2022.19459 36279114

[20] Osorio-de-Castro CGS Azeredo TB Pepe VLE Lopes LC Yamauti S Godman B. Policy change and the national essential medicines list development process in brazil between 2000 and 2014: has the essential medicine concept been abandoned? Basic Clin Pharmacol Toxicol. (2018) 122:402–12. 10.1111/bcpt.12932 29117642

[21] Brhlikova P Persaud N Osorio-de-Castro CGS Pollock AM. Essential medicines lists are for high income countries too. BMJ. (2023) 382:e076783. 10.1136/bmj-2023-076783 37669797

