# Supplementary material for: Access and use of WHO essential medicines in Italy
Source: Front Public Health. 2023 Oct 10;11:1211208. doi: 10.3389/fpubh.2023.1211208 (PMC10595003; doi:10.3389/fpubh.2023.1211208)
Supplement: Supplementary file 1 [file Table_1.docx]

**Table S.1**. Items of WHO EML (22^th^ edition) excluded from the analysis (n=18)

| EML section | | Items |
| --- | --- | --- |
| Medicines for reproductive health and perinatal care | Barrier methods | Condoms |
|  |  | Diaphragms |
|  | Intrauterine devices | Copper-containing device |
| Blood and its derivatives | Blood and blood components | Red blood cells |
|  |  | Whole blood |
|  |  | Fresh frozen plasma |
| Dermatological |  | Selenium sulfide |
|  |  | Urea |
| Gastrointestinal medicines | Oral rehydration | Oral rehydration salts |
|  |  | Oral rehydration salts - zinc sulfate |
| Antiseptics and disinfectants | Disinfectants | Alcohol based hand rub |
|  |  | Chloroxylenol |
|  |  | Glutaral |
| Vitamins and minerals |  | Ergocalciferol |
|  |  | Multiple micronutrient powder |
|  |  | Multiple micronutrient supplement |
| Dental preparations |  | Glass ionomer cement |
|  |  | Silver diamine fluoride |

**Table S.2**. List of EMs not authorised in Italy but imported as a foreign medicine in 2021 (n=47)

| EML section | | Active ingredient |
| --- | --- | --- |
| General anaesthetics and oxygen | Inhalational medicines | Halothane |
| Medicines for pain and palliative care | Medicines for other common symptoms in palliative care | Cyclizine |
| Antidotes and other substances used in poisonings |  | Succimer |
|  |  | Dimercaprol |
|  |  | Potassium ferric hexacyanoferrate (II) 2H2O |
|  |  | Fomepizole |
| Anti-infective medicines | Access group antibiotics | Phenoxymethylpenicillin |
|  |  | Procaine benzylpenicillin |
|  | Reserve group antibiotics | Polymyxin B (injection) |
|  | Antipneumocystosis and antitoxoplasmosis medicines | Sulfadiazine |
|  | Antituberculosis medicines | Streptomycin (injection) |
|  |  | Cycloserine |
|  |  | Rifapentine |
|  |  | Isoniazid + rifapentine |
|  |  | Ethionamide |
|  |  | Clofazimine |
|  | Antiamoebic and antigiardiasis medicines | Diloxanide |
|  | Antimalarial medicines | Primaquine |
|  |  | Pyrimethamine |
|  |  | Amodiaquine – sulfadoxine + pyrimethamine |
|  |  | Artemether |
|  |  | Artesunate |
|  |  | Artemether + lumefantrine |
|  | American trypanosomiasis | Benznidazole |
|  | Antileishmaniasis medicines | Meglumine antimoniate |
|  |  | Sodium stibogluconate |
|  |  | Miltefosine |
|  | Medicines for the treatment of 2nd stage African trypanosomiasis | Nifurtimox |
|  | Medicines for the treatment of 1st stage African trypanosomiasis | Suramin sodium |
|  | Intestinal anthelminthics | Praziquantel |
|  |  | Ivermectin |
|  | Antischistosomals and other antitrematode medicines | Triclabendazole |
|  | Antifilarials | Diethylcarbamazine |
| Cardiovascular medicines | Antiarrhythmic medicines | Lidocaine |
|  | Antihypertensive medicines | Hydralazine |
| Diuretics | Diuretics | Amiloride |
|  | Uterotonics | Ergometrine |
| Medicines for endocrine disorders | Adrenal hormones and synthetic substitutes | Fludrocortisone |
|  | Thyroid hormones and antithyroid medicines | Propylthiouracil |
|  |  | Iodine - Lugol's solution |
| Ophthalmological | Anti-infective agents | Natamycin |
| Diagnostic agents |  | Meglumine iotroxate |
| Blood products of human origin and plasma substitutes | Human immunoglobulins | Anti-rabies immunoglobulin |
| Muscle relaxants (peripherally-acting) and cholinesterase inhibitors |  | Vecuronium |
| Immunologicals | Sera, immunoglobulins and monoclonal antibodies | Anti-rabies virus monoclonal antibodies |
|  |  | Antivenom immunoglobulin |
| Vaccines | Recommendations for some high-risk populations | Dengue vaccine |

**Table S.3.** List of EMs not available in Italy in 2021 (n=25)

| **EML section** | | **Active ingredient** |
| --- | --- | --- |
| **Anti-infective medicines** | Access group antibiotics | Cloxacillin |
|  |  | Spectinomycin |
|  | Reserve group antibiotics | Plazomicin |
|  | Antituberculosis medicines | Ethambutol + isoniazid + pyrazinamide + rifampicin |
|  |  | Ethambutol + isoniazid + rifampicin |
|  | Antimalarial medicine | Amodiaquine |
|  |  | Amodiaquine – sulfadoxine + pyrimethamine |
|  |  | Artesunate + mefloquine |
|  |  | Artesunate + amodiaquine |
|  |  | Artesunate + pyronaridine tetraphosphate |
|  | Medicines for the treatment of 1st stage African trypanosomiasis | Fexinidazole |
|  | Medicines for the treatment of 2nd stage African trypanosomiasis | Melarsoprol |
|  |  | Eflornithine |
|  | Antischistosomals and other antitrematode medicines | Oxamniquine |
|  | Intestinal anthelminthics | Levamisole |
|  | Medicines for hepatitis C | Daclatasvir  Daclatasvir+sofosbuvir |
| **Immunomodulators and antineoplastics** | Cytotoxic medicines | Realgar-indigo naturalis formulation |
| **Cardiovascular medicines** | Thrombolytic medicines | Streptokinase |
| **Ophthalmological** | Anti-infective agents | Erythromycin |
|  | Anti-infective agents | Prednisolone |
|  | Mydriatics | Epinephrine |
| **Medicines for reproductive health and perinatal care** | Injectable hormonal contraceptives | Estradiol cypionate + medroxyprogesterone ac. |
|  |  | Norethisterone enantate |
|  |  | Medroxyprogesterone acetate |

**Table S.4.** List of EMs commonly marketed in Italy but not reimbursed in 2021 (n=18)

| **EML section** | | **Active ingredient** |
| --- | --- | --- |
| **Medicines for pain and palliative care** | Medicines for other common symptoms in palliative care | Docusate sodium |
|  | Non-opioids and non-steroidal anti-inflammatory medicines | Paracetamol |
| **Anti-infective medicines** | Antifungal medicines | Clotrimazole |
|  |  | Potassium iodide |
| **Medicines affecting the blood** | Antianaemia medicines | Ferrous salt + folic acid |
| **Gastrointestinal medicines** | Medicines for diarrhoea | Zinc sulfate |
| **Dermatological** | Antifungal medicines | Miconazole |
|  | Anti-infective medicines | Mupirocin |
|  | Anti-inflammatory and antipruritic medicines | Hydrocortisone |
| **Medicines for reproductive health and perinatal care** | Contraceptives | Levonorgestrel-releasing intrauterine system |
|  |  | Ethinylestradiol + etonogestrel |
|  |  | Ethinylestradiol + levonorgestrel |
|  |  | Levonorgestrel |
|  |  | Ulipristal |
|  |  | Norethisterone |
| **Medicines for mental and behavioural disorders** | Medicines for disorders due to psychoactive substance use | Nicotine replacement therapy |
| **Vitamins and minerals** |  | Thiamine |
|  |  | Riboflavin |

**Table S.5**. List of EMs available in Italy as galenic formulations, but not retrieved from the national administrative database in 2021 (n=11)

| **EML section** | | **Active ingredient** |
| --- | --- | --- |
| **Antidotes and other substances used in poisonings** | | Sodium nitrite |
| **Anti-infective medicines** | Antileprosy medicines | Dapsone |
| **Dermatological medicines** | Scabicides and pediculicides | Benzyl benzoate |
|  | Anti-infective medicines | Potassium permanganate |
|  | Medicines affecting skin differentiation and proliferation | Salicylic acid |
|  |  | Coal tar |
| **Cardiovascular medicines** | Antihypertensive medicines | Sodium nitroprusside |
| **Medicines for endocrine disorders** | Hyroid hormones and antithyroid medicines | Potassium iodide |
| **Ophthalmological** | Local anaesthetics | Tetracaine |
|  | Anti-infective agents | Tetracycline |
| **Ear, nose and throat medicines** |  | Acetic acid |

**Table S.6.** Expenditure and consumption of the main 15 subgroups belonging to “others” group

| Subgroup and active ingredient | Expenditure  (million euros) | %  cum | DDD  (million) | Average cost per DDD (euros) |
| --- | --- | --- | --- | --- |
| Antineoplastic agents (n=125) | 2,212 | 26.5 | 27 | 80.26 |
| Daratumumab | 239 | 11 | 1,59 | 150.03 |
| Osimertinib | 147 | 17 | 0,9 | 148.33 |
| Pertuzumab | 144 | 24 | 1,2 | 112.39 |
| Ruxolitinib | 110 | 29 | 0,9 | 110.85 |
| Palbociclib | 102 | 34 | 1,4 | 68.69 |
| Nintedanib | 78 | 38 | 1,0 | 76.34 |
| Dabrafenib | 65 | 41 | 0,6 | 105.73 |
| Trastuzumab emtansin | 63 | 44 | 0,3 | 193.74 |
| Ribociclib | 62 | 47 | 0,9 | 68.02 |
| Alectinib | 60 | 50 | 0,3 | 162.41 |
| Atezolizumab | 59 | 53 | 0,5 | 107.94 |
| Olaparib | 57 | 56 | 0,4 | 132.20 |
| Others (N=113) | 1,020 | 100 | 16,9 | 60.23 |
| Immunosuppressants and immunomodulators (n=38) | 1,080 | 39.4 | 41,9 | 25.8 |
| Eculizumab | 127 | 12 | 0,1 | 767.0 |
| Ustekinumab | 121 | 23 | 6,7 | 17.9 |
| Secukinumab | 111 | 33 | 3,6 | 30.6 |
| Vedolizumab | 70 | 40 | 2,1 | 32.6 |
| Abatacept | 67 | 46 | 1,3 | 49.4 |
| Tocilizumab | 60 | 52 | 2,3 | 25.5 |
| Canakinumab | 58 | 57 | 0,3 | 157.1 |
| Pirfenidone | 57 | 62 | 0,8 | 64.4 |
| Ixekizumab | 56 | 67 | 1,8 | 30.3 |
| Others (N=29) | 348 | 100 | 22,2 | 15.65 |
| Drugs for Multiple Sclerosis (n=11) | 715 | 47.9 | 24,3 | 29.3 |
| Dimethyl fumarate | 156 | 22 | 4,7 | 32.9 |
| Fingolimod | 137 | 41 | 2,5 | 53.9 |
| Natalizumab | 103 | 55 | 2,0 | 50.0 |
| Ocrelizumab | 102 | 69 | 2,1 | 48.3 |
| Others (N=7) | 15 | 100 | 12 | 17 |
| Blood glucose lowering drugs, excl. insulins (n=24) | 501 | 53.9 | 307 | 1.6 |
| Dulaglutide | 162 | 32 | 60 | 2.7 |
| Semaglutide | 73 | 47 | 4,3 | 16.8 |
| Linagliptin | 43 | 56 | 33 | 1.3 |
| Sitagliptin | 36 | 63 | 29 | 1.2 |
| Sitagliptin/metformin | 33 | 70 | 30 | 1.1 |
| Others (N=19) | 152 | 100 | 149 | 1.0 |
| Lipid-modifying agents (n=14) | 467 | 59.5 | 543 | 0.9 |
| Omega 3 | 123 | 26 | 107 | 1.1 |
| Ezetimibe | 92 | 46 | 126 | 0.7 |
| Ezetimibe/simvastatin | 71 | 61 | 109 | 0.7 |
| Ezetimibe/rosuvastatin | 57 | 73 | 126 | 0.5 |
| Evolocumab | 51 | 84 | 3,1 | 13.1 |
| Others (N=8) | 70 | 100 | 697 | 1.0 |
| Enzymatic Replacement Therapy (n=28) | 418 | 64.5 | 0,9 | 450.0 |
| Recombinant human acid alpha-glucosidase | 76 | 18 | 0,07 | 1.062.2 |
| Agalsidase alfa | 52 | 31 | 0,03 | 1.692.4 |
| Agalsidase beta | 45 | 42 | 0,09 | 483.6 |
| Imiglucerase | 45 | 53 | 0,04 | 1.096.5 |
| Idursulfase | 31 | 60 | 0,01 | 2.877.2 |
| Others (N=23) | 167 | 100 | 0,6 | 246.2 |
| Genito urinary system (n=10) | 269 | 67.7 | 0,8 | 0.32 |
| Systemic hormonal, excl. sex hormones and insulins (n=12) | 228 | 70.5 | 19 | 11.89 |
| Pain therapy (n=8) | 225 | 73.2 | 128 | 1.75 |
| Antihypertensives (n=11) | 223 | 75.8 | 372 | 0.60 |
| Cystic fibrosis (n=4) | 166 | 77.8 | 1 | 165.31 |
| Drugs for osteoporosis (n=7) | 157 | 79.7 | 130 | 1.20 |
| Asthma and chronic obstructive pulmonary disease (n=9) | 156 | 81.6 | 57 | 2.71 |
| Medicines for eye disorders (n=12) | 150 | 83.4 | 39 | 3.81 |
| Antivirals for SARS-CoV-2 (n=1) | 133 | 85.0 | 0,3 | 374.71 |
| Anti-parkinson drugs (n=11) | 126 | 86.5 | 62 | 2.0 |
| Antihemorrhagics (n=6) | 107 | 87.8 | 1 | 69.6 |
| Other drugs for disorders of musculo-skeletal system (n=4) | 97 | 88,9 | 0,7 | 138.5 |
| Dermatologicals (n=6) | 90 | 90.0 | 9 | 9.3 |
| Blood coagulation factors and other systemic hemostatics (n=4) | 79 | 91.0 | 0,1 | 677.1 |
| Antihypertensives for pulmonary arterial hypertension (n=4) | 77 | 91.9 | 1 | 44.3 |
| Biliar and hepatic therapy (n=7) | 72 | 92.8 | 59 | 1.2 |
| Diagnostic radiopharmaceuticals (n=54) | 68 | 93.6 | 0,1 | 455.4 |
| Antiepileptics (n=5) | 65 | 94.4 | 12 | 5.2 |
| Antiinflammatory and antirheumatic products, non-steroids (n=10) | 58 | 95.1 | 149 | 0.4 |
| Other hematological agents (n=6) | 33 | 95.5 | 0,032 | 1031.3 |
| Contrast media (n=9) | 27 | 95.8 | 0,5 | 50.8 |
| Drugs for peptic ulcer and gastro-oesophageal reflux disease (n=4) | 27 | 96.1 | 45 | 0.6 |
| Various (n=11) | 25 | 96.4 | 10 | 2.4 |
| Specific immunoglobulins and antiviral monoclonal antibodies (n=2) | 25 | 96.7 | 62 | 821.2 |
| Total first 30 subgroups (n=457) | **8,089** | **96.7** | **2,900** | **2.78** |
| Residual drugs (n=142) | 272 | 100 | 181 | 1.50 |
| Overall “others” group (n=599) | **8,362** | 100 | **3,081** | **2.71** |
